# Adequacy of Anesthesia Guidance Combined with Peribulbar Blocks Shows Potential Benefit in High-Risk PONV Patients Undergoing Vitreoretinal Surgeries

**DOI:** 10.3390/jcm14228081

**Published:** 2025-11-14

**Authors:** Dominika Majer, Michał J. Stasiowski, Anita Lyssek-Boroń, Katarzyna Krysik, Nikola Zmarzły

**Affiliations:** 1 Department of Ophthalmology, Prof. Kornel Gibiński Memorial University Clinical Centre, Medical University of Silesia, 40-752 Katowice, Poland; lekd.niedziela@gmail.com; 2 Chair and Department of Emergency Medicine, Faculty of Medical Sciences in Zabrze, Medical University of Silesia, 40-055 Katowice, Poland; 3 Department of Anaesthesiology and Intensive Care, St Barbara 5th Regional Hospital, Trauma Centre, 41-200 Sosnowiec, Poland; 4 Department of Ophthalmology, St Barbara 5th Regional Hospital, Trauma Centre, 41-200 Sosnowiec, Poland; anitaboron3@gmail.com (A.L.-B.); kkrysik@gmail.com (K.K.); 5 Department of Ophthalmology, Faculty of Medicine, Academy of Silesia, 40-055 Katowice, Poland; 6 Collegium Medicum, WSB University, 41-300 Dabrowa Gornicza, Poland; nikola.zmarzly@wsb.edu.pl

**Keywords:** postoperative nausea and vomiting, vitreoretinal surgery, peribulbar block, paracetamol, Adequacy of Anesthesia, oculocardiac reflex, oculoemetic reflex, numeric pain rating scale

## Abstract

**Background/Objectives**: Postoperative nausea and vomiting (PONV) are common after general anesthesia (GA) and, in patients undergoing vitreoretinal surgery, may be triggered by the oculocardiac reflex (OCR) leading to the oculoemetic reflex (OER). Inadequate dosing of intravenous rescue opioid analgesics may further provoke OCR. Adequacy of Anesthesia (AoA) monitoring enables optimized titration of intravenous rescue opioid analgesics, while preemptive intravenous or peribulbar analgesia may reduce opioid use. This study evaluated the impact of preemptive paracetamol or peribulbar block (PBB) combined with AoA-guided GA on the incidence of PONV, OCR, and OER in patients undergoing vitreoretinal surgery. **Methods**: A total of 185 patients were randomized to four groups: GA with AoA-guided intraoperative rescue opioid analgesia plus a single intravenous dose of paracetamol 1 g, or PBB using 1% ropivacaine, 0.5% bupivacaine, or a 1:1 mixture of 0.5% bupivacaine/2% lidocaine. Data from 175 patients were analyzed. **Results**: AoA-guided GA yielded an OCR incidence of 11.4% and PONV incidence of 4%. PBB, regardless of anesthetic solution, did not significantly reduce intraoperative rescue opioid analgesia requirements or the incidence of PONV, OCR, or OER compared with intravenous paracetamol. Notably, no PONV occurred in patients with three Apfel risk factors (predicted risk ≈ 61%) who received PBB. **Conclusions**: No overall advantage of PBB over intravenous paracetamol was observed. It may, however, benefit patients at high PONV risk.

## 1. Introduction

Postoperative nausea and vomiting (PONV) constitutes an adverse event following anesthesia [1]. Its prevention improves patient satisfaction [2,3], shortens hospital stay [4], and thereby enhances the cost-effectiveness of therapeutic procedures [5]. The incidence of PONV has been estimated at up to 60% after ophthalmic surgeries and up to 56% following vitreoretinal surgery [6]. Numerous anesthetic modalities are employed to reduce PONV, including multidrug PONV prophylaxis [7]; nonpharmacological methods such as acupressure [8], aromatherapy with peppermint [9] or essential oils [10], oral ginger intake [11], music therapy [12], and allowing patient visits [13]. Among these, the avoidance of intravenous opioids is considered most efficient [14], usually achieved by performing regional anesthesia alone [15].

Currently, vitreoretinal surgeries, including pars plana vitrectomy or phacovitrectomy, are most often carried out under regional anesthesia with monitored anesthesiological care. However, general anesthesia (GA) remains necessary in patients expected to have limited cooperation during lengthy procedures, particularly those with neurological deficits or at risk of respiratory disorders [16]. Intraoperative rescue opioid analgesics are administered when increases in heart rate or blood pressure suggest insufficient analgesia. Nevertheless, intraoperative rescue opioid analgesia has been identified as an independent risk factor for PONV after vitreoretinal surgery [17]. To minimize this risk, preventive analgesia (PA), administered either intravenously or regionally, is introduced prior to GA to reduce intraoperative rescue opioid analgesia requirements [18,19,20]. This strategy aims to improve hemodynamic stability, decrease PONV [18], reduce inappropriate postoperative pain perception (IPPP) [21], and limit the occurrence of oculocardiac and oculoemetic reflexes (OCR and OER) [18], although complete prevention remains elusive.

Surgical maneuvers inevitably evoke nociceptive stimulation. If intraoperative analgesia (antinociception) is inadequate, excessive stress hormone release may trigger hemodynamic instability. Underdosing of intravenous rescue opioid analgesics can result in IPPP [22] via central sensitization mechanism [23], whereas overdosing may cause hemodynamic collapse, requiring aggressive fluid therapy and vasoactive medications. Importantly, insufficient intravenous rescue opioid analgesic dosing is not always reflected by elevated heart rate or blood pressure, as volatile anesthetics may blunt hemodynamic responses to nociception in elderly patients with comorbidities [24]. Consequently, digital monitoring of the nociception–antinociception balance using surgical pleth index (SPI) has gained popularity [25]. SPI has been demonstrated to guide intravenous rescue opioid analgesic administration more effectively than reliance on hemodynamic changes alone [26,27,28]. Its clinical applicability is supported by several advantages: simple derivation from finger photoplethysmography [29], correlation with serum intravenous rescue opioid analgesics concentration [30], and ease of real-time monitoring (0 = effective antinociception, 100 = poor antinociception) [31]. Moreover, SPI-guided anesthesia has been associated with reduced cumulative intraoperative rescue opioid analgesia requirements and lower IPPP expressed in numeric pain rating scale (NPRS) compared with standard practice [32,33]. When combined with response entropy (RE) and state entropy (SE), which reflect depth of anesthesia, SPI constitutes the concept of Adequacy of Anesthesia (AoA).

To the best of our knowledge, no study has yet evaluated the potential effects of PA using an intravenous cyclooxygenase-3 (COX-3) inhibitor compared with peribulbar blocks (PBBs) with different local anesthetic mixtures under AoA guidance during vitreoretinal surgery.

A previous study of primary outcomes compared the effects of PA with either PBBs (using different local anesthetic mixtures) or a single intravenous dose of paracetamol (1 g) under AoA-guided GA on IPPP incidence, hemodynamic stability, and intraoperative rescue opioid analgesia requirements [34]. The aim of the current study was to analyze the incidence of OCR, PONV, and OER (secondary outcome measures) in these patients.

## 2. Materials and Methods

This study is one of two papers originating from the same clinical trial (Silesian MUKOAiIT8, NCT03413371), each addressing a distinct research question. The previously published paper is as follows: *Evaluating the Efficacy of Pre-Emptive Peribulbar Blocks with Different Local Anesthetics or Paracetamol Using the Adequacy of Anesthesia Guidance for Vitreoretinal Surgeries: A Preliminary Report. Biomedicines*. 2024;12(10):2303 [34]. The methodology for assessing IPPP in this study was based on our previous work [35], while the evaluation of PONV, OCR, and OER incidence was similarly adapted from earlier studies [36,37]. Similarly to previously published studies on AoA guidance of GA in vitreoretinal surgery, the already published primary outcome measures were the incidence of IPPP and hemodynamic stability [35], while the currently analyzed secondary outcome measures were the incidence of PONV, OCR, and OER.

Patients eligible for elective primary vitreoretinal surgery at the Department of Ophthalmology, St. Barbara 5th Regional Hospital in Sosnowiec, Poland, who met the inclusion criteria, were recruited. A total of 185 patients with an American Society of Anesthesiologists score of I–III participated in this prospective, randomized, controlled trial after providing written informed consent. Randomization was performed using sealed envelopes, assigning patients into four equal groups. This was performed by an assistant ophthalmologist (K.K.), who either performed PBB or administered intravenous paracetamol. The main anesthesiologist (M.S.), the operator (A.L-B.), and especially the post-anesthesia care unit (PACU) team (D.M.) were blinded to the group allocation. The study protocol was approved by the Bioethical Committee of the Medical University of Silesia in accordance with the Declaration of Helsinki (approval number KNW/0022/KB1/121/17, 5 December 2017; Chairman: Dr. Bogusław Okopien, Polish National Consultant for Pharmacy and Clinical Pharmacology). The trial was also registered in the Clinical Trial Registry (Silesian MUKOAiIT8, NCT03413371).

Exclusion criteria included: pregnancy; drug or alcohol abuse; history of allergy to local anesthetics or paracetamol; cardiac arrhythmia on ECG that could affect SPI monitoring; neurological disease or prior neurosurgical procedure that could interfere with entropy EEG monitoring; symptoms suggesting difficult laryngeal mask placement; pulmonary disease associated with risk of bronchospasm; history of acute or chronic pain; and anti-platelet therapy, which constitutes a contraindication to PBB.

During the preoperative visit, patients were informed about the possibility of IPPP. They were instructed on the use of the 10-point NPRS to report pain intensity, where 0 indicated no pain and 10 represented the worst pain imaginable [35,37,38,39,40].

### 2.1. Anesthesia Technique

All patients fasted for at least 12 h to reduce risk of diabetic gastropathy. On the day of surgery, prior to anesthesia induction, they received a single intravenous dose of midazolam (3.75–7.5 mg, Polfa Warszawa S.A., Warsaw, Poland), adjusted for age and body weight [41].

Upon arrival in the operating theater, patients in the bupivacaine/lidocaine (BL) group received preventive topical analgesia consisting of three instillations of 2% proparacaine (Sandoz/Novartis, Alcon Laboratories, Fort Worth, TX, USA). This was followed by a PBB using 3.5 mL of 2% lidocaine (Polfa Warszawa S.A., Warsaw, Poland) mixed with 3.5 mL of 0.5% bupivacaine (Polfa Warszawa S.A., Warsaw, Poland), administered by Hamilton’s technique 15 min before the induction of GA [42].

In the bupivacaine (BPV) group, after topical analgesia via triple instillation of 2% proparacaine, patients underwent PBB with 7 mL of 0.5% bupivacaine via Hamilton’s technique, 15 min before GA induction [42].

For the ropivacaine (RPV) group, the same topical regimen was used (three instillations of 2% proparacaine), followed by PBB with 7 mL of 0.75% ropivacaine (Molteni Farmaceutici, Scandicci, Italy) via Hamilton’s technique, again 15 min prior to GA induction [42].

Patients of the paracetamol (P) group received GA 30 min before entering the operating room, along with preemptive analgesia consisting of a single dose of 1 g paracetamol (Fresenius Kabi, Warsaw, Poland) diluted in 100 mL saline [42].

Just before surgery, all patients underwent five minutes of preoxygenation with 100% oxygen and were given Ringer’s solution intravenously (10 mL/kg body weight, B. Braun Melsungen AG, Melsungen, Germany). Anesthesia induction was achieved with fentanyl (FNT, 1 µg/kg, Polpharma, Warsaw, Poland) followed by propofol (2.5 mg/kg body weight, Fresenius Kabi GmbH, Bad Homburg, Germany), titrated to SE of 40–45 as guided by the previous studies [40,43,44,45].

After loss of consciousness, rocuronium (0.6 mg/kg, Esmeron, Fresenius, Warsaw, Poland) was administered, and after 45 s, a laryngeal mask airway was inserted. End-tidal CO_2_ was maintained at 35–37 mmHg, and anesthesia was maintained using sevoflurane, adjusted to keep SE within 35–45, in line with prior studies on vitreoretinal surgeries [40].

Throughout the procedure, standard monitoring included electrocardiography, heart rate, non-invasive blood pressure, oxygen saturation, fraction of inspired oxygen, end-tidal CO_2_, minimal alveolar concentration of sevoflurane, and fractions of inspired and expired sevoflurane. Depth of anesthesia was monitored via entropy EEG (SE and RE), and intraoperative analgesia was guided by the SPI, Adequate muscle relaxation was maintained with neuromuscular transmission monitoring (Carescape B650, GE Healthcare, Helsinki, Finland).

Detailed operational steps of the anesthesia protocol are presented in Appendix A.

### 2.2. Statistical Analysis

Sample size estimation was performed using G*Power (ver. 3.1.9.7; Heinrich Heine University, Düsseldorf, Germany) [46]. Based on a one-way ANOVA, the required sample size was calculated as 180 patients. Ultimately, data from 175 of the 185 enrolled patients were included in the analysis. A post hoc power calculation indicated that this sample size provided a power of 0.79.

Statistical analyses were performed using STATISTICA (ver. 13.3, StatSoft, Kraków, Poland). Continuous variables are presented as mean ± standard deviation and as median with interquartile range. Data distribution was assessed using the Shapiro–Wilk test. For comparisons between groups, the Kruskal–Wallis test was applied, followed by Dunn’s post hoc test. Categorical variables are reported as percentages and compared using the Chi-square test, or Fisher’s exact test when expected frequencies were <5. Bonferroni correction was applied for multiple comparisons. A *p*-value < 0.05 was considered statistically significant.

## 3. Results

**Figure 1:**
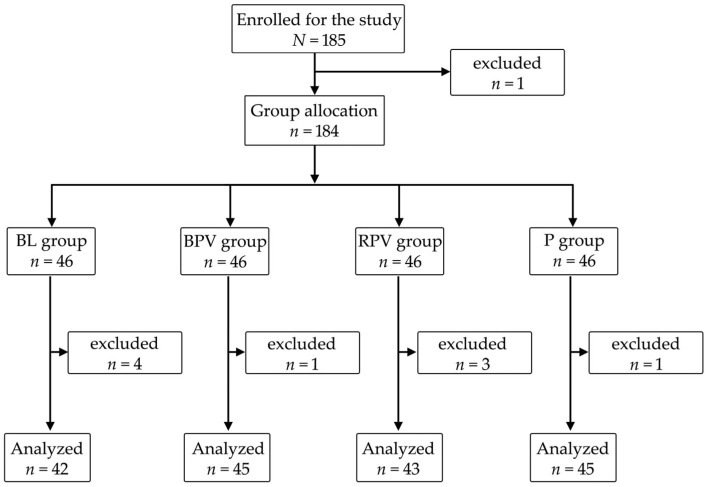
Patient enrollment and randomization flow chart. From 185 screened patients, 175 completed the protocol; exclusions were due to refusal of block, monitoring failure, postoperative agitation, inability to report pain, or preference for general anesthesia only.

A total of 185 patients were initially enrolled in the study [34]. One patient declined participation during the preoperative examination. The remaining 184 participants were randomized equally into four groups (*n* = 46 each: BL, BPV, RPV, and P). Nine patients were subsequently excluded: refusal to undergo PBB at the sight of a needle (*n* = 3), technical failure of intraoperative SPI measurement (*n* = 2), postoperative agitation that prevented observation during Stage 4 (*n* = 2), inability to report postoperative pain (*n* = 2), and preference for GA only (*n* = 1). Thus, 175 patients were included in the final analysis (99 women, 56.6%; 76 men, 43.4%; Figure 1).

As reported in our previous study [34], baseline demographic characteristics (age, height, weight, or BMI) did not differ significantly among the study groups (Table S1). The duration of phacovitrectomy was longer in the BL group compared with the BPV group, and the lowest maximum NPRS score was noted in the BPV group (Table S2).

In the present analysis, no significant differences were found in the incidence of postoperative adverse events across the study groups (Table 1).

No significant differences were found among study groups in Apfel score, sex, history of motion sickness, smoking status, or postoperative opioid use (Table 2).

A greater number of patients had one or two distinct PONV risk factors compared with those having zero or three (95% CIs for differences: 1 vs. 0, 24–41%; 2 vs. 0, 29–46%; 1 vs. 3, 28.7–44.5%; 2 vs. 3, 33–49%). However, no significant differences in the incidence of PONV were observed according to the number of risk factors. When stratified by study group, a significant difference in the distribution of Apfel scores was observed in the P group, driven by the absence of low-risk patients (score 0) and a higher proportion of patients with two risk factors (BPV vs. P, 95% CIs: 0, 5–26%; 2, −47–−7%; RPV vs. P, 95% CIs: 0, 4–24%; 2, −50–−10%). In contrast, combining the BL, BPV, and RPV groups (peribulbar block cohort) yielded a distribution similar to the overall population. No patients had an Apfel score of 4, as postoperative opioid analgesics were not administered (Table 3).

Further analysis was conducted to evaluate the relationship between PONV incidence, irrespective of group allocation, and the number of multivariate PONV risk factors reported in the current literature. No significant differences were observed among the analyzed factors (Table 4).

## 4. Discussion

Incidences of PONV remain a significant concern in surgical patients, negatively affecting postoperative outcomes [47] and causing sufficient distress that some patients are willing to pay additional costs to avoid it [3]. In the case of PONV after vitreoretinal surgery, an additional risk of suprachoroidal hemorrhage must be taken into account [48].

The incidence of PONV in patients undergoing vitreoretinal surgery is multifactorial. Volatile anesthetics have been shown to triple the incidence of PONV compared with intravenous hypnotics, whereas standard antiemetic prophylaxis using two drugs with different mechanisms of action can reduce it from 56% to 18% [6]. Adding regional blocks to GA in vitreoretinal surgery to reduce intraoperative rescue opioid analgesia has been reported to reduce the incidence of adverse events such as PONV, OCR, and OER [18], an effect particularly pronounced in pediatric patients [49,50]. Local anesthetics used in PBB act by inactivating sodium channels in their open state, thereby inhibiting fast sodium ion influx and reversibly blocking action potential propagation. Ropivacaine is a long-acting amide local anesthetic, and it selectively affects A, B, and C nociceptive fibers with lower cardiotoxicity and neurotoxicity compared to bupivacaine and lidocaine [51]. Paracetamol, a widely used non-opioid analgesic, exerts both central (COX-3 inhibition) and peripheral mechanisms of action [52,53,54,55,56,57], demonstrating efficacy in postoperative pain control [35,58,59,60,61,62,63], reducing PONV incidence [59,61,64], and maintaining a favorable safety profile [60,62,63,65], particularly in patients with gastrointestinal or renal comorbidities [60,66,67].

In the current study, neither intravenous paracetamol nor PBB significantly influenced overall PONV incidence, consistent with the lack of effect on IPPP and hemodynamic stability reported as primary outcomes [34]. Overall, PONV was observed in 4% of patients (7/175), distributed as follows: BL group, 2.38% (1/42); BPV group, 4.44% (2/45); RPV group, 9.3% (4/43); P group, 0%. One patient in the BL group experienced both nausea and vomiting, and one patient in the RPV group experienced retching. While no further complications occurred, dry retching may be particularly hazardous in vitreoretinal surgery, as PONV and intraoperative photocoagulation have been associated with suprachoroidal hemorrhage, with reported incidence up to 0.8% [48].

The low overall incidence of PONV compared with a 56% baseline risk of PONV, which can be reduced to 18% with multi-drug antiemetic prophylaxis [6], as well as other adverse events in our cohort, may be attributed to AoA guidance of intraoperative rescue opioid analgesia during GA. Precise titration under SPI guidance ensures proper nociception–antinociception balance, optimized fluid management, and depth of anesthesia, resulting in smooth postoperative recovery. Similar findings were reported by Feng et al. [68] in SPI-guided opioid-free anesthesia and Morel et al. [69] using bispectral index-guided GA with or without PBB in vitreoretinal surgery. In our study, higher intravenous crystalloid requirements in the RPV and P groups did not translate into higher PONV incidence. It is consistent with Sadrolsadat et al. [70], who observed no incidence of nausea in the PACU among patients undergoing vitreoretinal surgery who received paracetamol upon emergence from GA. This effect may be partially explained by systemic vascular resistance impairment induced by local anesthetics, as we previously observed in patients receiving continuous thoracic epidural anesthesia during open lumbar infrarenal aortic aneurysm repair under AoA-guided GA [71], where epidural ropivacaine led to hemodynamic instability compared to intravenous metamizole or tramadol. Additionally, underhydration combined with preoperative morning fasting represents an independent factor that can increase the risk of PONV through stimulation of serotonin secretion [72]. Therefore, maintaining balanced mesenteric perfusion by preventing gut ischemia or edema, and avoiding overhydration that could reduce serotonin release, may further contribute to reducing the incidence of PONV [73].

A similarly low incidence of PONV was observed in patients undergoing vitreoretinal surgery under GA with AoA guidance, either alone or combined with various intravenous or regional preventive analgesia (PA) techniques [37], as well as in patients receiving PA with COX-3 inhibitors [36], or in those undergoing endoscopic sinus surgery under total intravenous anesthesia (TIVA) with AoA guidance [74]. The literature also reports low PONV rates in vitreoretinal surgery, although these studies often included multidrug prophylaxis. For example, Heinke et al. [75] achieved a PONV incidence of only 4.7% using TIVA combined with antiemetic prophylaxis, comparable to the findings of the current study, despite our patients receiving volatile anesthetics and intravenous rescue opioid analgesics, both recognized independent risk factors for PONV [76]. Similarly, Reibaldi et al. [77] reported no PONV in 95.96% of patients undergoing vitreoretinal surgery under monitored anesthesiological care without intraoperative rescue opioid analgesia, who received ondansetron and dexamethasone prior to surgery with PBB using 1% ropivacaine (analogous to the RPV group in the present study). In contrast, single-drug prophylaxis in comparable patients resulted in PONV rates between 20–30%.

According to the current guidelines, baseline prophylaxis with two antiemetic drugs (e.g., dexamethasone combined with a 5-HT3 receptor antagonist, like in the current study) is recommended, with additional agents for high-risk patients [78]. Assessment of risk factors, including female sex, younger age, surgery type, motion sickness, non-smoking status, prior PONV, anesthesia duration >2 h, postoperative opioid use, volatile anesthetics, as well as regimens reducing patient’s baseline risk (e.g., via regional anesthesia or employing non-opioid analgesics), are advocated [3,78,79,80,81]. Intravenous opioid analgesia, particularly continuous postoperative oxycodone, can induce nausea in up to 94% and vomiting in 26% of patients [82], highlighting the importance of intraoperative rescue opioid analgesia-sparing strategies. Therefore, we assume that AoA guidance may herald a new era in PONV prevention in patients undergoing vitreoretinal surgery, possibly replacing multi-drug prophylaxis, changing the anesthetic regimen to digital GA monitoring, along with risk stratification of PONV using established scales.

In this study, the Apfel score was used as the most widely accepted method for risk stratification for predicting PONV [79]. PONV incidence across Apfel risk scores was as follows: 1/15 (8.33%) for zero risk factors, 4/72 (5.55%) for one risk factor, 2/80 (2.5%) for two risk factors, and 0/8 for three risk factors. Notably, all high-risk patients were in groups receiving PBB, while none were in the P group. This suggests that AoA-guided GA combined with PBB may effectively prevent PONV in high-risk patients, whereas intravenous paracetamol alone did not achieve similar outcomes [36]. The pharmacological mechanism of action of local anesthetics in reducing PONV may involve some currently unknown effects on the central nervous system, similar to those achieved with intravenous infusion of lidocaine as an adjunct to GA [83], which has been shown to cross the blood/brain barrier, resulting in a significant reduction in the incidence of PONV and IPPP [84]. This should be balanced against potential risk of performance of PBB, including local anesthetic systemic toxicity (LAST) [85]. Standard treatment for LAST includes airway management, adequate oxygenation, seizure control, and lipid emulsion therapy, which is now routinely used for LAST. Therefore, it is important to remember that lipid emulsion therapy should always be readily available to treat potential LAST after PBB [86].

Currently, liberal PONV prophylaxis is recommended in both adult and pediatric populations, even without formal risk stratification [7]. However, in this study, among 167 patients with zero, one, or two risk factors (low predicted risk), PONV occurred in only seven cases (4.16%). A similar observation was made by previously mentioned Reibaldi et al. [77]. Likewise, in our recent study evaluating intravenous PA with COX-3 inhibitors in patients undergoing vitreoretinal surgery under AoA-guided GA, PONV was observed in only 3/142 patients (2.11%) with low risk [36]. These findings raise the question of whether applying current prophylaxis guidelines universally to patients with multiple cardiovascular comorbidities undergoing vitreoretinal surgery may cause more harm than benefit, given the potential complications of antiemetic drugs. For instance, ondansetron may prolong the QT interval even at low doses in healthy individuals [87], while the use of dexamethasone remains controversial [88,89,90]. Further studies in larger cohorts are required to determine whether a universal multimodal prophylaxis strategy is still preferable over a risk-adapted approach based on validated scoring systems [91] together with digital GA monitoring in patients undergoing vitreoretinal surgery, taking into account the safety of postoperative recovery, free from unnecessary polypharmacy with unpredictable consequences. Finally, in this study, additional risk factors such as diabetes mellitus (both insulin-dependent and non-insulin-dependent), obesity, underweight, and IPPP did not correlate with PONV incidence, although previous studies have identified them as independent predictors in patients undergoing different surgical procedures [38,92,93]. We hypothesize that AoA-guided GA may have attenuated not only the potential impact of preoperative PBB or paracetamol on PONV incidence across study groups, but also the influence of additional risk factors previously reported in the literature.

PONV can also be evoked by the OCR, a trigeminal–vagal reflex triggered by pressure on the eyeball or traction of extraocular muscles, which may also provoke the OER [94,95]. OCR is identified by a rapid decrease in heart rate of more than 20% [96], which can occasionally progress to severe bradyarrhythmia with hemodynamic collapse. This phenomenon is most commonly recognized in the pediatric population undergoing ophthalmic procedures [97,98]. The reported incidence of OCR is as high as 70%, with variability depending on the type of surgery and the anesthetic technique employed, particularly the use of regional anesthesia, which provides effective intraoperative antinociception [18,99].

In the present analysis, OCR was observed in 20 of 175 patients (11.43%): 5 patients in the BL group (11.9%), 6 in the BPV group (13.33%), 4 in the RPV group (9.3%), and 5 in the P group (11.11%). These differences were not statistically significant. We concluded that the potential influence of PA on OCR was likely blunted by effective antinociception achieved through AoA guidance. This contrasts with the findings of Ghali et al. [18], who reported 27% OCR incidence with standard GA.

In our view, the precise administration of intravenous rescue opioid analgesics only in cases of insufficient antinociception, identified by ∆SPI > 15 compared with stage 2 values, likely contributed to stable FNT serum concentrations, which changes are known to correlate with SPI fluctuations [30]. This strategy may have suppressed central sensitization [23], a process implicated in triggering IPPP, and thereby contributed to the low OCR incidence. Preventing central hyperexcitability [23] in turn may have helped to reduce both PONV and OER.

Several limitations of the present analysis must be acknowledged. First, IPPP, which is a subjective phenomenon [99], and nausea are burdensome in quantification and can be underreported. Mild nausea may have been misinterpreted by some patients as general discomfort following GA and vitreoretinal surgery. Second, IPPP beyond discharge from PACU to Department of Ophthalmology was not recorded, as the study aimed specifically to correlate intraoperative SPI values with IPPP during stage 4. Moreover, patient behaviors such as arousal, rapid position changes, or coughing were reported to interfere with SPI monitoring [100], making such comparison clinically irrelevant. Moreover, the study being conducted in a single center limits the possibility of generalizing the obtained results, which requires further multi-center studies in this area. In addition, a post hoc power analysis indicated a statistical power of 0.79, slightly below the commonly recommended threshold of 0.80. Although this difference is small, it may nonetheless limit statistical sensitivity. Finally, both single-dose paracetamol and single-shot PBB are known to last for approximately six hours, restricting the clinical relevance of later NPRS values and late PONV. Post-discharge PONV and adverse events were not assessed due to rapid patient discharge of majority of patients in the peri-pandemic period. Relationships between intraoperative maneuvers and adverse events will be analyzed separately due to word count limitations.

## 5. Conclusions

In conclusion, the current analysis of secondary outcomes did not demonstrate any advantage of either intravenous paracetamol (1 g) or single-shot PBB, regardless of the local anesthetic mixture used, administered prior to vitreoretinal surgery, in reducing the incidence of OCR, OER, or PONV under AoA-guided GA, despite the overall low incidence of adverse events. Any potential benefit of PBB in high-risk individuals warrants further investigation.

## Figures and Tables

**Table 1:** Rate of postoperative adverse events by study group.

Intraoperative Data *N* (%)	Total *N* = 175 (100%)	BL Group *n* = 42 (24%)	BPV Group *n* = 45 (25.7%)	RPV Group *n* = 43 (24.6%)	P Group *n* = 45 (25.7%)	*p*-Value	95% CI (BL/BPV/RPV/P)
PONV in the PACU (early PONV)	7 (4%)	1 (2.9%)	2 (4.4%)	4 (9.3%)	0 (0%)	0.1 NS	0.1–12.6/0.5–15.2/2.6–22.1/0–7.9
Nausea in the PACU (early nausea)	2 (1.1%)	1 (2.4%)	0 (0%)	1 (2.3%)	0 (0%)	0.4 NS	0.1–12.6/0–7.9/0.1–12.3/0–7.9
Vomiting in the PACU (early vomiting)	6 (3.4%)	1 (2.4%)	2 (4.4%)	3 (7%)	0 (0%)	0.3 NS	0.1–12.6/0.5–15.2/1.5–19.1/0–7.9
Retching in the PACU (early severe unproductive vomiting)	1 (0.6%)	0 (0%)	0 (0%)	1 (2.3%)	0 (0%)	0.5 NS	0–8.4/0–7.9/0.1–12.3/0–7.9
Both nausea and vomiting in the PACU (early nausea and vomiting/retching)	2 (1.1%)	1 (2.4%)	0 (0%)	1 (2.3%)	0 (0%)	0.4 NS	0.1–12.6/0–7.9/0.1–12.3/0–7.9
IPPP (acute + moderate pain perception)	18 (10.2%)	5 (11.9%)	1 (2.2%)	6 (14%)	6 (13%)	0.2 NS	4–25.6/0.1–12.9/5.3–27.9/5.1–26.8
OCR	20 (11.4%)	5 (11.9%)	6 (13.3%)	4 (9.3%)	5 (11.1%)	0.97 NS	4–25.6/5.1–26.8/2.6–22.1/3.7–24.1
OER (OCR + PONV)	0 (0%)	0 (0%)	0 (0%)	0 (0%)	0 (0%)	-	0–8.4/0–7.9/0–8.2/0–7.9

BL—bupivacaine/lidocaine; BPV—bupivacaine; RPV—ropivacaine; P—paracetamol; Sd—standard deviation; Me—median; IQR—interquartile range; PONV—postoperative nausea and vomiting; PACU—post-anesthesia care unit; IPPP—inappropriate postoperative pain perception; OCR—oculocardiac reflex; OER—oculoemetic reflex; NS—not significant.

**Table 2:** Distribution of postoperative nausea and vomiting (PONV) risk factors across study groups.

Intraoperative Data	Total *N* = 175 (100%)	BL Group *n* = 42 (24%)	BPV Group *n* = 45 (25.7%)	RPV Group *n* = 43 (24.6%)	P Group *n* = 45 (25.7%)	*p*-Value	95% CI (BL/BPV/RPV/P)
Apfel score X ± Sd Me (IQR)	1.49 ± 0.7 2 (1)	1.5 ± 0.7 1.5 (1)	1.4 ± 0.7 1 (1)	1.4 ± 0.9 1 (1)	1.7 ± 0.5 2 (1)	0.1 NS	1–2/1–2/1–2/2–2
Apfel (%) X ± Sd Me (IQR)	0.3 ± 0.12 0.4 (0.2)	0.3 ± 0.12 0.3 (0.2)	0.3 ± 0.12 0.2 (0.2)	0.3 ± 0.15 0.2 (0.2)	0.3 ± 0.1 0.4 (0.2)	0.07 NS	0.2–0.4/0.2–0.4/0.2–0.4/0.4–0.4
Sex Female/Male *n* (%)	99/76 56.57%/43.4	26/16 61.9%/38.1%	23/22 51.1%/48.9%	19/24 44.2%/55.8%	31/14 68.9%/31.1%	0.09 NS	45.6–76.4/35.8–66.3/29.1–60.1/53.4–81.8
Motion sickness Yes/No *n* (%)	11/164 93.7%/6.3%	2/40 4.8%/95.2%	2/43 4.4%/95.6%	5/38 11.6%/88.4%	2/43 4.4%/95.6%	0.5 NS	0.6–16.2/0.5–15.2/3.9–25.1/0.5–15.2
History of PONV Yes/No *n* (%)	0/175 0%/100%	0/42 0%/100%	0/45 0%/100%	0/43 0%/100%	0/45 0%/100%	-	0–8.4/0–7.9/0–8.2/0–7.9
Smoking Yes/No *n* (%)	25/150 14.3%/85.7%	7/35 16.7%/83.3%	7/38 15.6%/84.4%	9/34 20.9%/79.1%	2/43 4.4%/95.6%	0.1 NS	7–31.4/6.5–29.5/10–36/0.5–15.2
Postoperative use of opioid drugs Yes/No *n* (%)	0/175 0%/100%	0/42 0%/100%	0/45 0%/100%	0/43 0%/100%	0/45 0%/100%	-	0–8.4/0–7.9/0–8.2/0–7.9

BL—bupivacaine/lidocaine; BPV—bupivacaine; RPV—ropivacaine; P—paracetamol; Sd—standard deviation; Me—median; IQR—interquartile range; PONV—postoperative nausea and vomiting; NS—not significant.

**Table 3:** Apfel score distribution and its relationship with postoperative nausea and vomiting (PONV).

Apfel Score [Point] *N* (%)	0 (10% Risk of PONV)	1 (21% Risk of PONV)	2 (39% Risk of PONV)	3 (61% Risk of PONV)	*p*-Value	95% CI
Total *N* = 175 (100%)	15 (8.6%)	72 (41.1%)	80 (45.7%)	8 (4.6%)	0 vs. 1, *p* < 0.001; 0 vs. 2, *p* < 0.001; 1 vs. 3, *p* < 0.001; 2 vs. 3, *p* < 0.001	1 vs. 0, 24–41; 2 vs. 0, 29–46; 1 vs. 3, 28.7–44.5; 2 vs. 3, 33–49
PONV *n* = 7 (4%)	1 (14.3%)	4 (57.1%)	2 (28.6%)	0 (0%)	0.6 NS	0 risk factors: 0.2–31.9 1 risk factor: 1.5–13.6 2 risk factors: 0.3–8.7 3 risk factors: 0–36.9
No-PONV *n* = 168 (96%)	14 (8.3%)	68 (40.5%)	78 (46.4%)	8 (4.8%)		
BL group	2 (4.8%)	19 (45.2%)	19 (45.2%)	2 (4.8%)	BPV vs. P, *p* = 0.03; RPV vs. P, *p* = 0.02	BPV vs. P: 0, 5–26; 1, −9–31; 2, −47–−7; 3, −6–6; RPV vs. P: 0, 4–24; 1, −12–29; 2, −50–−10; 3, −3–17
BPV group	7 (15.6%)	20 (44.4%)	17 (37.8%)	1 (2.2%)		
RPV group	6 (13.9%)	18 (41.9%)	15 (34.9%)	4 (9.3%)		
P group	0 (0%)	15 (33.3%)	29 (64.4%)	1 (2.2%)		
BL + BPV + RPV group	15 (11.5%)	57 (43.9%)	51 (39.2%)	7 (5.4%)	0 vs. 1, *p* < 0.001; 0 vs. 2, *p* < 0.001; 1 vs. 3, *p* < 0.001; 2 vs. 3, *p* < 0.001	1 vs. 0, 22–43; 2 vs. 0, 18–38; 1 vs. 3, 29–49; 2 vs. 3, 25–43

BL—bupivacaine/lidocaine; BPV—bupivacaine; RPV—ropivacaine; P—paracetamol; PONV—postoperative nausea and vomiting; NS—not significant.

**Table 4:** Distribution of multifactorial PONV risk factors in patients with and without postoperative nausea and vomiting (PONV).

Factors	PONV	No-PONV	*p*-Value	95% CI
Non-DM	6 (4.8%)	119 (95.2%)	0.7 NS	0.3–115.9
Overall DM	1 (2%)	49 (98%)		
Insulin-dependent	1 (3.4%)	28 (96.6%)	1.0 NS	0.02-inf
Insulin-independent	0 (0%)	21 (100%)		
Normal weight	1 (1.96%)	50 (98.04%)	0.6 NS	Normal: 0.05–10.5; Overweight: 1.6–14.2; Obese: 0.5–14.3
Overweight	4 (5.8%)	65 (94.2%)		
Obesity	2 (4%)	46 (96%)		
IPPP	0 (%)	18 (100%)	1.0 NS	0–6.3

DM—diabetes mellitus; PONV—postoperative nausea and vomiting; IPPP—inappropriate postoperative pain perception; NS—not significant.

## Data Availability

The original contributions presented in this study are included in the article/Supplementary Material. Further inquiries can be directed to the corresponding author(s).

## Supplementary Materials

The following supporting information can be downloaded at: https://www.mdpi.com/article/10.3390/jcm14228081/s1, Table S1: Anthropometric characteristics of patients across study groups; Table S2: Comparison of postoperative pain incidence and severity between study groups.

## Abbreviations

The following abbreviations are used in this manuscript:

AoA	Adequacy of Anesthesia
BL	Bupivacaine/lidocaine
BPV	Bupivacaine
COX-3	Cyclooxygenase-3
DM	Diabetes mellitus
FNT	Fentanyl
GA	General anesthesia
IPPP	Inappropriate postoperative pain perception
MAP	Mean arterial pressure
NPRS	Numeric pain rating scale
OCR	Oculocardiac reflex
OER	Oculoemetic reflex
P	Paracetamol
PA	Preventive analgesia
PACU	Post-anesthesia care unit
PBB	Peribulbar block
PONV	Postoperative nausea and vomiting
RE	Response entropy
RPV	Ropivacaine
SE	State entropy
SPI	Surgical pleth index

## Appendix A. Detailed Anesthesia Protocol

### Appendix A.1. Stage 1

At the time of admission to the operating theater, monitoring devices were applied in accordance with the manufacturers’ instructions. EEG entropy electrodes (for RE and SE) were positioned on the patient’s forehead, while a pulse oximeter for SPI monitoring was placed on the finger contralateral to the venous access site. The non-invasive blood pressure cuff was attached to the right arm, and standard ECG electrodes were applied to the patient’s back. Baseline measurements of all monitored parameters were then recorded.

All procedures involving PBB were performed by the ophthalmic surgeon (K.K.), who has more than 15 years of experience in vitreoretinal surgeries.

### Appendix A.2. Stage 2

During stage 2, SPI values were recorded beginning five minutes after laryngeal mask placement and continued until the start of orbital sterilization, allowing sufficient time for sensor calibration. The mean SPI value was then calculated from these measurements. Throughout this period, patients received a balanced crystalloid infusion at a rate of 5 mL/kg/h, with adjustments made according to individual requirements.

### Appendix A.3. Stage 3—Intraoperatively

The SPI was continuously monitored in real time, with values recorded at one-minute intervals. When the SPI exceeded the mean Stage 2 value by more than 15 points, patients received an intravenous rescue dose of FNT (1 µg/kg body weight). Doses could be repeated every five minutes until the SPI returned to baseline. The duration of vitreoretinal surgery was defined as the interval between speculum placement and removal.

Initially, it was assumed that an FNT dose of 1 μg/kg would provide adequate analgesia prior to speculum insertion. Previous findings by Gruenewald et al. indicated that ΔSPI > 10 or an absolute SPI > 50 may predict insufficient analgesia [25], whereas other studies have suggested that an absolute SPI threshold of >50 should guide rescue opioid administration [101]. To minimize the risk of FNT overdosing due to misinterpretation of SPI fluctuations, we adopted a stricter protocol: rescue analgesia was administered only when ΔSPI > 15 compared with the Stage 2 baseline and sustained for at least one minute.

All vitreoretinal surgeries were carried out by the ophthalmic surgeon (A.L.-B.), who has over 10 years of experience and performs more than 400 procedures annually.

OCR was monitored and defined as a sudden reduction in heart rate of 20% or more from baseline during ocular manipulation. If OCR occurred, the surgeon temporarily stopped the procedure. When it persisted, 0.5 mg of atropine (Polfa Warszawa S.A., Warsaw, Poland) was administered intravenously. Moreover, 0.5 mg atropine dose was given if the heart rate fell below 45 beats per minute.

Intraoperative hypotension, defined as mean arterial pressure (MAP) < 65 mmHg, was initially treated with a bolus of balanced crystalloids (5 mL/kg). If MAP did not normalize, 10 mg of intravenous ephedrine (Polfa Warszawa S.A., Warsaw, Poland) was administered. In cases of hypertension (MAP > 110 mmHg) with adequate SPI values, intravenous urapidil (Takeda Pharma, Vienna, Austria) was given until MAP returned to the normal range.

### Appendix A.4. Stage 4—Postoperatively

Patients were monitored in the PACU by a different anesthesiological team led by the first author (D.M.), who was not involved in the surgical or anesthetic procedures during stages 1–3 and therefore was blinded to group allocation, ensuring unbiased assessment. Postoperative monitoring included SPI, MAP, heart rate, diastolic arterial pressure, systolic arterial pressure, and arterial oxygen saturation. In addition, patients were observed for adverse events such as PONV, sedation, allergic reactions, and pain for 24 h.

For the prevention and treatment of PONV, patients received an intravenous dose of 4 mg ondansetron (Accord Healthcare Limited, London, UK). In cases of hypotension (MAP < 65 mmHg), Optilyte solution (500 mL, Fresenius Kabi, Warsaw, Poland) was infused at 5 mL/kg body weight. Supplemental oxygen (3 L/min) was delivered via nasal cannula.

Pain was evaluated every 10 min using the NPRS. When it exceeded 3, patients received an individualized rescue dose of either a non-steroidal anti-inflammatory drug or metamizole, in line with current guidelines of the Polish Society of Anaesthesiologists [22]. SPI values were continuously recorded, with mean values logged at one-minute intervals (CARESCAPE Monitor B650, software ver. 3.1). Pain intensity was categorized as mild (NPRS 0–3), moderate (NPRS 4–6), or acute (NPRS 7–10).

Patients remained in the PACU for at least 30 min before transfer to the Department of Ophthalmology. At this point, monitoring was discontinued, except for cases involving PONV, which were documented throughout the first 24 h postoperatively. Each episode of nausea was treated with 4 mg dexamethasone (Jelfa, Jelenia Góra, Poland), while vomiting was additionally managed with 4 mg ondansetron (Accord Healthcare Limited, London, UK).

Discharge to the DoVS was permitted once four conditions were fulfilled: pain at rest < 4 on NPRS, MAP > 65 mmHg, HR between 60–90 bpm, and Aldrete score > 8.
