# Supplementary material for: Adequacy of Anesthesia Guidance Combined with Peribulbar Blocks Shows Potential Benefit in High-Risk PONV Patients Undergoing Vitreoretinal Surgeries
Source: J Clin Med. 2025 Nov 14;14(22):8081. doi: 10.3390/jcm14228081 (PMC12653265; doi:10.3390/jcm14228081)
Supplement: Supplementary file 1 [file jcm-14-08081-s001.zip › Table S1.pdf]

Journal of Clinical Medicine

MDPI

Table S1. Anthropometric characteristics of patients across study groups.

Metrics		Total N = 175 (100%)	BL group *n* = 42 (24%)	BPV group *n* = 45 (25.7%)	RPV group *n* = 43 (24.6%)	P group *n* = 45 (25.7%)	*p*-Value
Age X ± Sd Me (IQR)	[years]	66.1 ± 9.5<br>67 (11)	66.3 ± 11.4<br>66.5 (13)	66.29 ± 7.6<br>68 (8)	66.23 ± 9.7<br>68 (12)	65.24 ± 9.6<br>66 (9)	0.83<br>NS
Gender *n* (%)	Female	99 (56.57)	26 (61.9)	23 (51.1)	19 (44.2)	31 (68.9)	0.09<br>NS
	Male	76 (43.4)	16 (38.1)	22 (48.9)	24 (55.8)	14 (31.1)	
Height X ± Sd Me (IQR)	[cm]	166.2 ± 9<br>165 (13)	165.7 ± 8.3<br>164.5 (10)	168.9 ± 9<br>168 (11)	166.7 ± 9.94<br>168 (18)	163.7 ± 8.1<br>163 (11)	0.07<br>NS
Weight X ± Sd Me (IQR)	[kg]	77.4 ± 14.4<br>76 (20)	77.3 ± 16.1<br>73 (14)	80.3 ± 13.3<br>79 (21)	78.1 ± 15<br>76 (19)	74.2 ± 13.1<br>74 (19)	0.22<br>NS
BMI X ± Sd Me (IQR)	[kg/m^2]	28 ± 4.7<br>27.7 (5.9)	28.2 ± 5.5<br>26.8 (4.8)	28 ± 3.3<br>27.5 (4.9)	28.1 ± 4.7<br>28.1 (7)	27.8 ± 5<br>27.9 (7)	0.86<br>NS
BMI *n* (%)	underweight	0 (0%)	0 (0%)	0 (0%)	0 (0%)	0 (0%)	0.6<br>NS
	norm	51 (30%)	12 (29.3%)	9 (22%)	14 (32.6%)	16 (35.6%)	
	overweight	69 (40.6%)	20 (48.8%)	19 (46.3%)	15 (34.9%)	15 (33.3%)	
	obesity	50 (29.4%)	9 (22%)	13 (31.7%)	14 (32.6%)	14 (31.1%)	

BL—bupivacaine/lidocaine; BPV—bupivacaine; RPV—ropivacaine; P—paracetamol; Sd—standard deviation; Me—median; IQR—interquartile range; BMI—body mass index; NS—not significant.
