# Supplementary material for: Adequacy of Anesthesia Guidance Combined with Peribulbar Blocks Shows Potential Benefit in High-Risk PONV Patients Undergoing Vitreoretinal Surgeries
Source: J Clin Med. 2025 Nov 14;14(22):8081. doi: 10.3390/jcm14228081 (PMC12653265; doi:10.3390/jcm14228081)
Supplement: Supplementary file 1 [file jcm-14-08081-s001.zip › Table S2.pdf]

Journal of Clinical Medicine

MDPI

Table S2. Comparison of postoperative pain incidence and severity between study groups.

Postoperative pain		Total N = 175 (100%)	BL group n = 42 (24%)	BPV group n = 45 (25.7%)	RPV group n = 43 (24.6%)	P group n = 45 (25.7%)	p-Value
Time of surgery X ± Sd Me (IQR)	Pars plana vitrectomy [min]	47.9 ± 20.8<br>43.5 (33)	54.9 ±27<br>52 (48)	40.9 ± 14.5<br>41 (22)	38.3 ± 15.5<br>33 (24)	53.1 ± 14.6<br>51 (23)	0.1<br>NS
	Phacovitrectomy [min]	53.2 ± 16.6<br>49 (23)	62.8 ± 18.7<br>59.5 (24)	47.9 ± 15.5<br>45 (17)	51.2 ±15.1<br>44 (23)	52.2 ± 18.1<br>50.5 (22)	BL vs. BPV, $p = 0.01$
FNT X ± Sd Me (IQR)	[μg]	121.1 ± 76.8<br>100 (100)	131 ± 92.1<br>100 (150)	116.7 ± 64.8<br>100 (100)	111.4 ± 65.3<br>100 (100)	122.4 ± 80.3<br>100 (100)	0.97<br>NS
Intraoperative fluid therapy X ± Sd Me (IQR)	mL	1082.85 ± 309.42<br>1000 (350)	1097.56 ± 270.64<br>1000 (250)	1057.21 ± 284.14<br>1000 (450)	1234.39 ± 355.48<br>1250 (500)	940 ± 254.75<br>1000 (250)	BL vs. P, $p = 0.04$;<br>RPV vs. P, $p = 0.0003$
NPRS max X ± Sd Me (IQR)	[1÷10]	0.87 ± 1.76<br>0 (0)	0.95 ± 1.78<br>0 (1)	0.09 ± 0.6<br>0 (0)	1.19 ± 2.07<br>0 (2)	1.27 ± 1.97<br>0 (2)	BPV vs. P, $p = 0.03$
Type of first pain perception n (%)	Mild	157 (89.7%)	37 (88.1%)	44 (97.8%)	37 (86.1%)	39 (86.7%)	0.3<br>NS
	Moderate	16 (9.1%)	5 (11.9%)	1 (2.2%)	5 (11.6%)	5 (11.1%)	
	Acute	2 (1.1%)	0 (0%)	0 (0)	1 (2.3%)	1 (2.2%)	

BL—bupivacaine/lidocaine; BPV—bupivacaine; RPV—ropivacaine; P—paracetamol; Sd—standard deviation; Me—median; IQR—interquartile range; FNT—fentanyl; NPRS—numerical pain rating scale; NS—not significant.

https://doi.org/10.3390/jcm14228081
